# HLA-B*57:01 Complexed to a CD8 T-Cell Epitope from the HSV-2 ICP22 Protein Binds NK and T Cells through KIR3DL1

**DOI:** 10.3390/v14051019

**Published:** 2022-05-11

**Authors:** Kerry J. Laing, Victoria L. Campbell, Lichun Dong, David M. Koelle

**Affiliations:** 1 Department of Medicine, University of Washington, Seattle, WA 98195, USA; vlc32@uw.edu (V.L.C.); lichun.dong@maxvax.cn (L.D.); dkoelle@medicine.washington.edu (D.M.K.); 2 Department of Laboratory Medicine and Pathology, University of Washington, Seattle, WA 98195, USA; 3 Vaccine and Infectious Diseases Division, Fred Hutchinson Cancer Center, Seattle, WA 98109, USA; 4 Department of Global Health, University of Washington, Seattle, WA 98195, USA; 5 Benaroya Research Institute, Seattle, WA 98101, USA

**Keywords:** CD8 T cell, T-cell epitope, NK cell, KIR3DL1, HLA-B*57:01, herpes simplex virus

## Abstract

HLA-B*57:01 is an HLA allelic variant associated with positive outcomes during viral infections through interactions with T cells and NK cells, but severe disease in persons treated with the anti-HIV-1 drug abacavir. The role of HLA-B*57:01 in the context of HSV infection is unknown. We identified an HLA-B*57:01-restricted CD8 T-cell epitope in the ICP22 (US1) protein of HSV-2. CD8 T cells reactive to the HSV-2 ICP22 epitope recognized the orthologous HSV-1 peptide, but not closely related peptides in human IFNL2 or IFNL3. Abacavir did not alter the CD8 T-cell recognition of the HSV or self-derived peptides. Unexpectedly, a tetramer of HSV-2 ICP22 epitope (228–236) and HLA-B*57:01 bound both CD8 T cells and NK cells. Tetramer specificity for KIR3DL1 was confirmed using KIR3DL1 overexpression on non-human primate cells lacking human KIR and studies with blocking anti-KIR3DL1 antibody. Interaction with KIR3DL1 was generalizable to donors lacking the HLA-B*57:01 genotype or HSV seropositivity. These findings suggest a mechanism for the recognition of HSV infection by NK cells or KIR-expressing T cells via KIR3DL1.

## 1. Introduction

Class I HLA molecules are fundamentally important in the immune control of viral infections by activating CD8 T cells and interacting with natural killer (NK) cells. The class I allelic variant HLA-B*57:01 is classified as a Bw4 family member and has been associated with improved disease outcomes for HIV/AIDS. The superior virologic control of HIV-1 is associated with the HLA-B*57:01 genotype [1], HLA-B*57:01-restricted CD8 T-cell responses [2], and specific T-cell receptor (TCR) sequence attributes [3]. At the same time, the expression of the killer cell immunoglobulin-like receptor (KIR), KIR3DL1—an inhibitory receptor on NK cells—in HLA-B*57:01 positive persons is associated with the elite control of HIV-1 and, potentially, increased NK function [4,5].

HLA-B*57:01 is also associated with drug hypersensitivity reactions, particularly to abacavir, a medication used to treat HIV-1 [6]. Alterations in HLA-bound peptide conformation have been hypothesized as abacavir can bind in the peptide-binding groove of HLA-B*57:01. This may elicit inappropriate CD8 T-cell activity that manifests itself as severe hypersensitivity. The effect is dependent on the properties of the peptide C-terminus, whereby peptides ending with small hydrophobic residues, such as isoleucine, leucine, and valine, potentially become neo-epitopes in the presence of abacavir. Peptides ending in large hydrophobic residues, such as phenylalanine or tryptophan, may also lose binding, and, as a consequence, the recognition of pathogen peptides may be reduced [7,8].

While peptides complexed with HLA class I activate T cells through TCR engagement, they can also interact with immune cells via KIR. There are at least 17 known functional members of the polymorphic KIR family. These can be activating (S type, for short cytoplasmic tail, containing an immunoreceptor-tyrosine activating motif (ITAM)) or inhibitory (L type, long cytoplasmic tail, containing an inhibitory ITIM motif) [9]. KIR are expressed by both NK cells and a subset of T cells. Inhibitory KIR reduce the activation of NK cells in the presence of their appropriate HLA ligand. Along with other HLA allelic variants sharing the Bw4 determinant, HLA-B*57:01 appears to have specificity for KIR3DL1. Most persons possess one or two functional copies of either *KIR3DL1*, [9,10,11] or *KIR3DS1*, an activating variant of *KIR3DL1* that can occur at the same location in the KIR locus on human chromosome 19 [12]. *KIR3DL1* has many allelic variants that encode different protein sequences with differing expression levels on the cell surface, which impacts the strength of NK cell inhibition [9,13]. The specificity of the interaction with HLA-B*57:01 may depend on the amino acid residue 166 of KIR3DL1 (which is a leucine in the common allele KIR3DL1*001) [14,15] and can be blocked with the KIR3DL1-specific monoclonal antibody (mAb) DX9. KIR3DS1 has a contrasting amino acid (arginine) at the corresponding residue shown to modulate HLA-Bw4 binding [15]. The interaction of HLA-B*57:01 with KIR3DL1/DS1 residue 166 is further influenced by the HLA-B*57:01-bound peptide, specifically peptide position 8 (P8). For example, KIR3DL1 binding is abrogated if epitope P8 is a negatively charged (aspartic acid or glutamic acid) amino acid and KIR3DS1 binding is favored if epitope P8 is an aromatic amino acid (tryptophan, phenylalanine or tyrosine) [16], consistent with a structural role for peptides in modulating HLA-KIR interactions.

NK cells are large CD3-negative lymphocytes that are defined phenotypically by the cell surface expression of CD56 and/or CD16. Both markers vary in expression depending on the function and level of differentiation of the NK cells. The two major populations of NK cells, defined as CD56^bright^ and CD56^dim^, represent less differentiated, higher-cytokine-producing NK cells and more differentiated, more cytotoxic NK cells, respectively [17]. More prevalent in blood than CD56^bright^ NK cells, CD56^dim^ NK cells are more likely to express KIR.

NK cells have long been recognized as being functionally important for host defense against herpesvirus infections, including HSV species in particular. In 1989, Biron et al. reported severe herpes infections in a patient with phenotypic NK cell absence [18], later related to mutation in *GATA2* [19]. Since then, several other rare mutations have been reported in persons with quantitative or qualitative NK cell deficiencies, which make subjects susceptible to herpesviruses (reviewed in [20]). A lower level of activating receptors on CD56^dim^ NK cells has been suggested as a potential correlate with HSV severity [21]. These reports emphasize the significance of defining how NK cells can sense HSV and mechanisms that could counteract HSV recognition.

During the study of HLA-B*57:01-restricted CD8 T cells that react to human herpesvirus-2, the major causative agent of genital herpes, we identified a viral peptide epitope that can activate CD8 T cells. Unexpectedly, a complex of this newly discovered HSV-2 epitope with HLA-B*57:01 also interacted with NK cells. We show that interaction with NK cells is mediated through KIR3DL1 binding, and that a sub-population of CD8 T cells, including from persons without HSV infection or HLA-B*57:01, can also bind to HLA-B*57:01-peptide via KIR3DL1.

## 2. Materials and Methods

### 2.1. Subjects and Specimens

Subjects seropositive for HSV-2 were recruited to the University of Washington Virology Research Clinic. The study was approved by the University of Washington Institutional Review Board (IRB STUDY00004400) and participants provided written informed consent. Class I HLA typing was performed at the Puget Sound Blood Center (now Bloodworks) or by PCR-based genotyping [22]. Peripheral blood mononuclear cells (PBMC) were isolated using Lymphoprep (Cosmo Bio USA, Carlsbad, CA, USA) and cryopreserved at 10 × 10^6^ cells/mL/vial in 10% DMSO (Fisher, Hampton, NH, USA), 40% human serum (Valley Biomedical, Winchester, VA, USA), and 50% RPMI-1640 (Hyclone, Logan, UT, USA). Epstein–Barr virus (EBV) strain B95-8 was used to immortalize B lymphocyte continuous lines (B-LCL) from PBMC [23]. HSV-1 and HSV-2 seropositivity was assessed by serum Western blot [24].

Bulk T-cell lines were generated from HSV-2 reactive CD8 T cells, as previously described [25]. In brief, subject-specific monocyte-derived dendritic cells were generated from adherent PBMCs using IL-4 and GM-CSF [26] and were combined with an equal number (2 × 10^5^) of UV-irradiated (Stratalinker XL1000, 180,000 μJ) HeLa cells that were uninfected (mock) or infected overnight with HSV-2 strain 186 (MOI 2.5). After incubation for 4 h at 37 °C/5% CO_2_, 1 × 10^6^ negative-selected (STEMCELL EasySep Human CD8 T Cell Isolation Kit) autologous CD8 T cells were added per well. T cells were harvested after 18–20 h of incubation and stained with anti–CD3-ECD (UCHT1, Beckman Coulter, Brea, CA, USA), anti–CD8-FITC (3B5, Thermo Fisher Scientific, Waltham, MA, USA), anti–CD137-APC (4B4-1, BD Biosciences, San Jose, CA, USA), and 7-AAD (BD Biosciences). Live (7-AAD negative), single CD3+ CD8+ CD137+ lymphocytes were sorted from HSV-2-exposed PBMC using a BD Biosciences FACS Aria II (University of Washington Cell Analysis Facility, Seattle, WA, USA).

Sorted cells were expanded by stimulation with 1.6 μg/mL phytohemagluttinin P (PHA, Remel, San Diego, CA, USA), in the presence of 2 × 10^6^/mL γ-irradiated (3300 rads) allogeneic PBMC, for 2–3 weeks (37 °C, 5% CO_2_) in a T-cell medium (TCM) containing RPMI-1640, 4% human serum, 4% defined fetal bovine serum (Hyclone, Logan, UT, USA), 2 mM L-glutamine (Hyclone), and 100 U/mL penicillin/streptomycin (Gibco). Natural IL-2 (nIL-2, 32 U/mL, Hemagen, Columbia, MD, USA) was added after 48 h and replenished with fresh TCM twice weekly. Polyclonal T-cell lines were further expanded using anti-CD3 as published [27] and the resultant T-cell lines were cryopreserved.

### 2.2. Antigens

Vero cells infected with HSV-2 (strain 186) stocks were harvested when cytopathic effect reached 80%, disrupted by sonication, and the cellular debris removed by low-speed centrifugation. The supernatant was aliquoted and stored at −80 °C. Each HSV-2 open reading frame (ORF) was cloned into Gateway^TM^ pDONR221 (Invitrogen, Grand Island, NY, USA), as previously described [28,29]. HSV-2 ORFs were subcloned to pDEST103 [30] for the eukaryotic intracellular expression of eGFP-HSV-2 fusion proteins.

Targeted HSV-2 ICP22 (*US1*) peptides (8–10 amino acids) were designed using the algorithmic prediction of HLA binding [31] (Table 1). Sequence variants for an immunogenic HSV2-US1 (228–236) peptide RTRLGPRTW were identified for HSV-1 (HSV1-US1 (232–240), RaRLaPRTW), and for human IFNL3 (IFNL3 (76–84), RsRLfPRTW) and human IFNL2 (IFNL2 (76–84), hsRLfPRTW) using BLASTP (BLAST^®^, NCBI) with search limits set to HSV-1 or human. Each HSV and human peptide was predicted to be an avid binder to HLA-B*57:01 [31] (not shown). Peptides (Sigma, >70% pure) were reconstituted in DMSO.

### 2.3. Identification of HLA-B*57:01 Restricted CD8 T-Cell Responses to HSV-2 Proteins

Artificial APC (aAPC) expressing HLA-B*57:01 and individual HSV-2 ORFs were used to detect CD8 T-cell responses in CD8 T-cell lines enriched for HSV-2 reactivity. Briefly, HSV-2 ORFs cloned into pDEST103 (100 ng/well) and empty p103 negative control plasmids were co-transfected, in duplicate, with pcDNA3.1 constructs encoding HLA-B*57:01 (100 ng/well) into COS-7 cells as previously described [30]. After 48 h, T cells (1 × 10^5^ in TCM) were added and co-cultures incubated for 24 h at 37 °C. PHA (1.6 µg/mL) were used as a positive control. Secreted IFN-γ in supernatants was measured by ELISA [30]. In brief, high binding ELISA plates (MICROLON™ 600) were coated overnight at 4 °C with mouse anti-human IFN-γ mAb (clone 2GI, Thermo Fisher, Waltham, MA, USA) and blocked with Tris-buffered saline (TBS) containing 0.1% bovine serum albumin (BSA) for 1 h at room temperature. Supernatants were added to blocked plates for 2 h at room temperature after washing with PBS-Tween (Dulbecco’s phosphate-buffered saline (PBS, Corning) containing 0.1% Tween-20 (Fisher Scientific, Waltham, MA, USA)), after the incubation of the plates for 2 h at room temperature. After washing, detection antibody (mouse anti-human IFN-γ-Biotin conjugate, clone B133.5, Thermo Scientific, Waltham, MA, USA) was added for 2 h at room temperature, plates washed, and avidin-horse radish peroxidase (HRP) added for 30 min at room temperature. TMB substrate (KPL) was added to the washed plates and reactions stopped after 10 min with 1M phosphoric acid (Fisher Scientific). Results are reported as absorbance (OD_450_) and determined using a Wallac Victor^3^ plate reader (Perkin Elmer, Waltham, MA, USA). Responses to HSV-2 proteins were considered positive if both OD_450_ values were greater than 0.15 and at least 2-fold higher than negative controls.

Epitope mapping in ICP22 was performed using autologous B-LCL as antigen-presenting cells. Equal numbers (1 × 10^5^) of B-LCL and CD8 T cells were combined with individual peptides in TCM and incubated at 37 °C for 20–24 h. Peptides were screened at a final concentration of 1 µg/mL each peptide and <0.3% DMSO. For titration, a log10 dilution series was used between 1 µg/mL and 0.1 pg/mL. An equivalent concentration of DMSO served as a negative control, while PHA (1.6 µg/mL) was used as a positive control. Medium alone (MED) controls were additionally included in some experiments. To investigate if HLA-B*57:01 restricted T-cell reactivity to HSV2-US1 (228–236) would be influenced by abacavir, T cells were combined with B-LCL that were pre-treated with abacavir (10 µg/mL) or left untreated for 24 h [32]. HSV2-US1 (228–236) and related variant peptides (Table 1) were added at 1 µg/mL, and cultures incubated at 37 °C for 24 h. Peptide-driven T-cell activation was determined by measuring IFN-γ in cell supernatants by ELISA [33]. The HSV2-US1 (228–236) epitope was uploaded to the Immune Epitope Database (Epitope ID 9063706).

### 2.4. Detection of T-Cell and NK-Cell Binding by B*57:01 Tetramer Containing HSV2-US1 (228–236)

To confirm the HLA-restriction of the HSV2-US1 (228–236) RTRLGPRTW peptide, a custom PE-conjugated peptide-HLA-B*57:01 tetramer (ImmunAware, Hørsholm, Denmark), termed B57-RTR, was tested for binding to HSV-2-specific CD8 T-cell lines and PBMC. The specificity of B57-RTR tetramer for binding KIR3DL1 was assessed using COS-7 cells that overexpressed KIR3DL1. In brief, 100 ng of either pReceiver-M02:KIR3DL1 (EX-A1726-M02, Genecopoeia) or pReceiver-M02:eGFP (negative control, EX-EGFP-M02, Genecopoeia) expression plasmids were transfected into COS-7 cells in 96-well plates using 0.5 µL Fugene 6 (Promega, Madison, WI, USA) as per the manufacturer’s instructions. After 48 h, trypsin-harvested cells were pre-incubated for 30 min at room temperature in FACS buffer (Dulbecco’s phosphate-buffered saline (PBS), 1% BSA, 0.01% NaN_3_) with 5 µg/mL mAb DX9 (Invitrogen, Waltham, MA, USA) or an isotype (mouse IgG1) control antibody (Biolegend, San Diego, CA, USA), washed, and then stained with B57-RTR (or mock stained with FACS buffer) for 30 min at room temperature. Binding was evaluated using a BD Canto II flow cytometer (University of Washington Cell Analysis Facility, Seattle, WA, USA) and FlowJo Version 10 (for Mac, BD, Franklin Lakes, NJ, USA).

Tetramers were also used to stain T-cell lines (3 × 10^5^) or PBMC (1 × 10^6^) for 30 min at room temperature in the dark, after staining for 20 min with LiveDead (Near-IR, Invitrogen) and before staining for 30 min with anti-CD3-ECD (UCHT1, Beckman Coulter, Burea, CA, USA), anti-CD8-FITC (3B5, Thermofisher, Waltham, MA, USA), anti-CD56-APC (HCD56, Biolegend, San Diego, CA, USA), and anti-CD16-PacificBlue (3G8, Biolegend, San Diego, CA, USA) in FACS buffer. For some experiments, anti-KIR3DL1-AlexaFluor700 antibody (DX9; Biolegend, San Diego, CA, USA) was included prior to staining with tetramer. For other experiments, cells were pre-incubated for 30 min with the unconjugated DX9 antibody or the isotype control antibody (as above), before staining with B57-RTR. Stained cells were fixed in PBS containing 1% formaldehyde. Data were acquired using a four-laser BD Canto II flow cytometer (University of Washington Cell Analysis Facility, Seattle, WA, USA) and analyzed using FlowJo Version 10.

## 3. Results

### 3.1. HLA-B*57:01-Restricted CD8 T Cells Recognize HSV-2 Proteins

**Figure 1:**
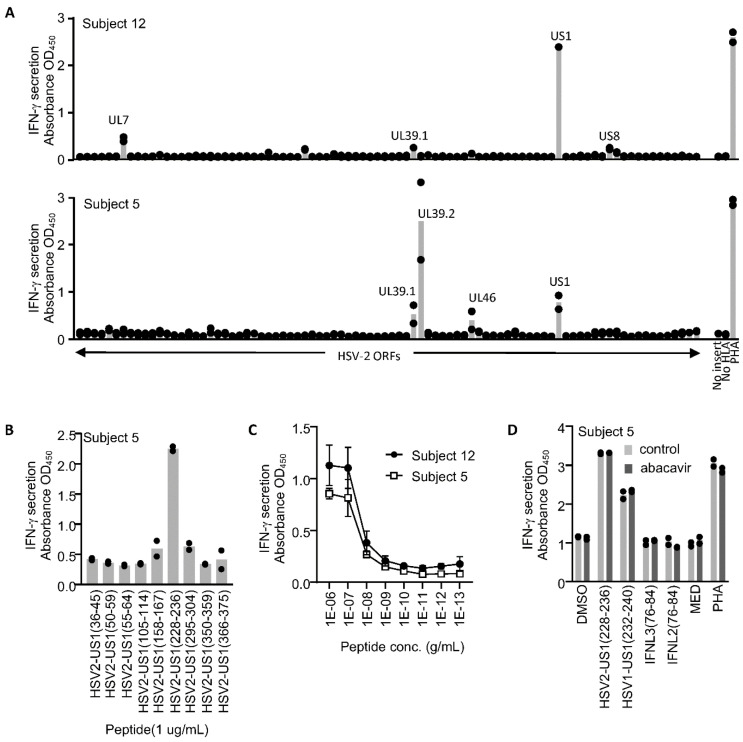
(**A**) T-cell lines generated from CD8 T cells activated (CD137^high^) following exposure to HSV-2 cross-presented antigen were screened for their protein specificity. An expression library representing all HSV-2 ORFs was co-transfected with HLA-B*57:01 into COS-7 cells to generate artificial antigen-presenting cells (aAPC). HSV-2-specific CD8 T-cell lines were exposed to aAPC and activation measured by IFN-γ secretion. HSV-2 ORFs that generated responses are labeled. (**B**) An epitope at HSV-2 ICP22 (ORF US1) aa 228–236 (sequence RTRLGPRTW; abbreviated RTR) was detected by testing predicted HLA-B*57:01 binding peptides. (**C**) RTR peptide was active to 10 ng/mL for T-cell lines from both subjects. (**D**) Exposure to abacavir (10 µg/mL) did not alter the recognition of RTR or the homolog from HSV-1 (HSV1-US1 (232–240)), and did not reveal activation in the presence of peptides with similar sequences in human IFNL3 (76–84) RsRLfPRTW or IFNL2 (76–84) hsRLfPRTW. MED, medium alone; PHA, phytohemagluttinin P. Alternative amino acids are shown in lower case. All tests were performed in duplicate with raw data indicated and means shown as bars (**A**,**B**,**D**) or with means shown as symbols and standard deviations shown as error bars (**C**).

HSV-2 specific CD8 T cells were isolated from the PBMC of two subjects with the HLA-B*57:01 genotype by selecting activated (CD137^high^) cells after HSV-2 antigen was cross-presented by monocyte-derived dendritic cells. The specific recognition of individual HSV-2 proteins presented by HLA-B*57:01 was determined by measuring IFN-γ responses to aAPC expressing only this HLA allele (Figure 1A). Several HSV-2 ORFs were antigenic. Both T-cell lines reacted to *US1* and *UL39*, while single subjects reacted to *UL7*, *US8*, and *UL46*. Responses to ICP22 (the protein product of ORF *US1*) were decoded using peptides predicted to bind HLA-B*57:01. This identified the amino acids 228–236, abbreviated RTR, as CD8 T-cell antigens for both subjects (Figure 1B,C). The peptide remained active at concentrations as low as 10 ng/mL (Figure 1C).

Since HLA-B*57:01 is associated with hypersensitivity reactions to abacavir, we assessed whether abacavir has any impact on CD8 T-cell activation by RTR, or by sequence-similar peptides from HSV-1 or human interferon lambda (Table 1). We observed the cross-recognition of the HSV-1 cognate peptide, but not the interferon-derived peptides. Abacavir did not alter peptide-driven responses (Figure 1D).

### 3.2. HLA-B*57:01:US1 Tetramer Binds to Both T Cells and NK Cells

**Figure 2:**
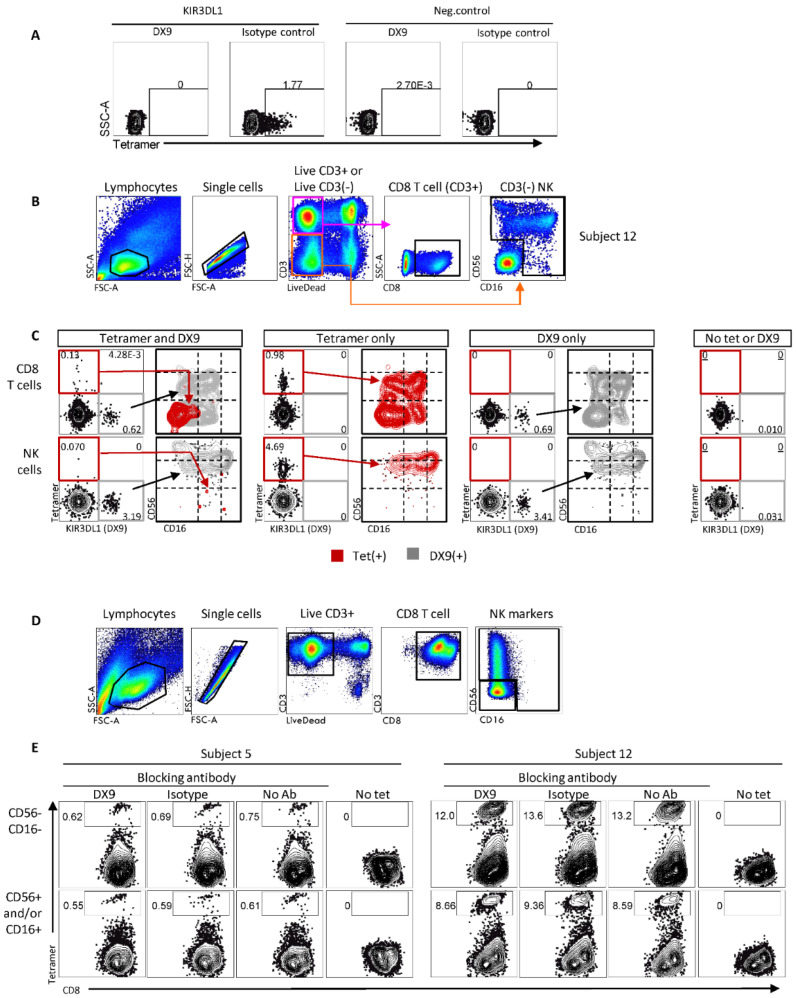
B57-RTR tetramer binds NK and T cells in a KIR3DL1-dependent and -independent manner. (**A**) B57-RTR tetramer binds COS-7 cells transfected with KIR3DL1, but not to cells transfected with a negative control expression plasmid. The interaction was blocked by anti-KIR3DL1 antibody DX9 but not an isotype control. (**B**) Gating scheme showing CD8 T cells (CD3^pos^CD8^pos^) and NK cells (CD3^neg^ and (CD56^pos^ and/or CD16^pos^)) subsets within PBMC lymphocytes. (**C**) B57-RPR tetramer binding of CD8 T cells and NK cells (from (**B**)) was compared in the presence or absence of DX9 fluorescently conjugated antibody. DX9 stained discrete subsets of both CD8 T and NK cells and largely inhibited tetramer binding. A modest frequency (0.13%) of CD8 T cells, but only a few NK cells, remained tetramer-positive in the presence of DX9. The CD56 and CD16 phenotypes for B57-RTR tetramer positive (red gate) and DX9-positive events (gray gate) are shown. Note: Tetramer or DX9 gated events are plotted using contour plots with outliers shown. In these overlay graphs, event frequencies are scaled within the population applied to a grey DX9 or red tetramer layer, and are thus are meaningful for comparing phenotypes but are not quantitative. “Tet” and “tetramer” refer to fluorescent B57-RTR tetramer. (**D**) Gating scheme to separate HSV-2-specific CD8 T-cell lines into CD56^neg^ and CD56^pos^ populations. (**E**) Both HSV-2-specific CD8 T-cell line subsets (from (**D**)) bound B57-RTR tetramer, but binding was not reduced by DX9, consistent with tetramer binding through TCR. Throughout, numbers indicate the percentage of cells in the indicated gate(s).

We constructed a tetramer of RTR and HLA-B*57:01 to confirm the HLA-B*57:01 restriction of the T-cell response to ICP22 (Figure 1). Unexpectedly, the B57-RTR tetramer was bound by both CD3^pos^ T cells, and by CD3^neg^ cells in PBMC from subject 12. To determine the phenotype of the CD3^neg^ cells, we co-stained PBMC with NK cell markers and observed the binding of B57-RTR to CD3^neg^ NK cells that were positive for the NK markers CD56 and/or CD16 (Figure 2).

Since other HLA-B*57:01-peptide ligands can bind KIR3DL1 on NK cells [34], we examined if the B57-RTR tetramer could bind to KIR3DL1. Tetramer binding to KIR3DL1 was confirmed by flow cytometry using COS-7 cells transfected with KIR3DL1, while COS-7 cells transfected with negative control (EGFP) expression plasmid did not bind B57-RTR (Figure 2A). Furthermore, tetramer binding was blocked by pre-incubating transfected COS-7 cells with anti-KIR3DL1 mAb DX9, but not with an isotype control antibody, supporting a direct KIR3DL1 interaction with the HLA-B*57:01:US1 peptide complex.

To further differentiate tetramer binding to KIR3DL1 and TCR, we probed PBMC from subject 12 with B57-RTR tetramer and AlexaFluor700-conjugated DX9 (Figure 2B,C). DX9, when used alone, showed about 0.7% of CD8 T cells and 3.4% of NK cells were KIR3DL1 positive (DX9 only plots, Figure 2C). Similarly, nearly 1% of CD8 T cells and 4.7% of NK cells bound B57-RTR when used alone (tetramer only plots, Figure 2C). Co-staining revealed that 0.13% of tetramer-positive CD8 T cells were DX9-negative, supporting an interaction with CD8 T cells that lack KIR3DL1, presumably via TCR (tetramer and DX9 plots, Figure 2C). These B57-RTR^pos^ DX9^neg^ CD8 T cells were CD56^neg^CD16^neg^, while most B57-RTR^neg^ DX9^pos^ CD8 T cells were CD56^dim^ and/or CD16^dim^, suggesting KIR3DL1 expression occurs primarily on T cells with an NK-cell-like phenotype. Only a few NK cells were B57-RTR^pos^ in the presence of DX9, consistent with binding of B57-RTR to NK cells mediated by KIR3DL1. When used alone, DX9^pos^ and B57-RTR^pos^ NK cells shared the same phenotypes—predominantly CD56^pos^ (dim and bright) with varying levels of CD16—consistent with binding to the same population of NK cells.

To evaluate whether B57-RTR tetramer binding to enriched, expanded HSV-2-specific CD8 T-cell lines was mediated by KIR3DL1 or TCR interactions, we preincubated CD8 T-cell lines from subjects 5 and 12 with DX9 or isotype control antibody before staining with tetramer. The evaluation of CD56 and CD16 was included to assess the expression of these markers in cell lines originally sorted as T cells (CD3-positive). Little CD16 expression was observed, but some CD8 T cells expressed CD56 (Figure 2D), consistent with an activated phenotype [35]. Only a small decrease in tetramer staining was observed in the presence of DX9 compared to isotype control in CD8 T cells without NK markers (10–12% reduction in binding) or with NK markers (7–8% reduction), supporting the CD8 T-cell recognition of B57-RTR through TCR rather than KIR3DL1 (Figure 2E).

### 3.3. KIR3DL1 Binding Is Independent from HLA-B*57:01 Genotype or HSV-2 Antigen Experience

**Figure 3:**
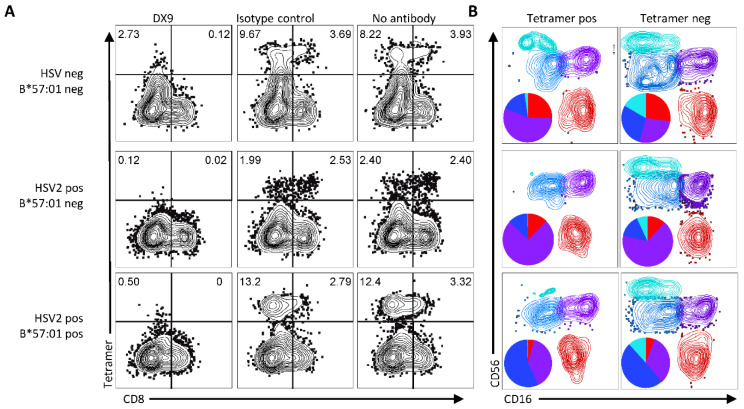
(**A**) B57-RTR tetramer binds gated NK cells in PBMC regardless of HLA type and HSV infection status in a KIR3DL1-dependent manner. Subject characteristics are displayed at left. Gating of NK cells as in Figure 2B. (**B**) Comparison of CD16 and CD56 expression between B57-RTR tetramer-positive and tetramer-negative NK cells from each donor. Contour plots show NK subpopulations of CD56^bright^CD16^neg^ (light blue), CD56^dim^CD16^neg/dim^ (dark blue), CD56^dim^CD16^bright^ (purple), and CD56^neg^CD16^bright^ (red) populations. Piecharts show relative proportions of each subpopulation within B57-RTR tetramer-positive or tetramer-negative NK cells in matching colors. Tetramer-positive NK cells had fewer CD56^bright^CD16^neg^ NK cells (light blue) relative to tetramer-negative NK cells.

We next tested whether HSV-2 infection or HLA-B*57:01 genotype were necessary for the interaction of B57-RTR with NK cells. NK cell binding was evaluated using PBMC of three subjects: 1) one HSV-seronegative subject without prior exposure to the HSV-2 RTR epitope, 2) one HSV-2 seropositive subject that did not possess the HLA-B*57:01 allele, and 3) HSV-2 seropositive subject 5 (Figure 1) from which the B57-RTR epitope was identified. Tetramer binding was assessed for cells pre-incubated with DX9, isotype control antibody, or no blocking antibody. NK cells were gated by flow cytometry as in Figure 2B. The B57-RTR tetramer bound >4% of NK cells from each subject, suggesting independence from HLA type or prior antigenic exposure (Figure 3A). DX9 blocked tetramer binding for all three subjects tested, confirming interaction with NK cell KIR3DL1. The phenotype of tetramer-positive NK cells from samples stained with no blocking antibody was compared to the tetramer negative cells. We observed that B57-RTR tetramer-positive NK cells had a lower proportion CD16^neg^CD56^bright^ cells than did the tetramer-negative cells (Figure 3B; Chi test, *p* < 0.0001 for all subjects). This is consistent with CD56^bright^ NK cells lacking KIR expression, since CD56^dim^ (KIR-suppressed) NK cells upregulate CD56 upon activation [17,35].

## 4. Discussion

The HLA-Bw4 allele HLA-B*B57:01 can be advantageous in the control of viral disease, as evidenced in detail for HIV-1, but is detrimental for persons receiving abacavir. Understanding the mechanism(s) by which HLA-B*57:01 benefits control of viral infections, whether through TCR-mediated activation of T cells or by tuning of HLA-KIR interactions, is of medical importance. In this paper, we show that HLA-B*57:01 complexed to a peptide epitope from the HSV-2 ICP22 protein (encoded by ORF *US1*) can activate CD8 T cells, and also bind with NK cells in a KIR3DL1-dependent manner. This indicates that the sensing of the same viral peptide may independently influence two cell populations to modulate HSV-2 infection.

This is the first known example of KIR3DL1 binding by an HLA-bound human herpesvirus peptide, but HLA-B*57:01 interactions are known for other viral systems. Several epitopes from HIV-1, including the TW10 epitope from HIV-1 Gag, when complexed with HLA-B*57:01 can bind KIR3DL1 [34]. A similar finding was described for an HLA-B*57:01-restricted CD8 T-cell epitope in dengue virus NS1 [36]. Circulating NK cells from persons with severe dengue that bound the B57-NS1 tetramer were activated ex vivo, consistent with a functional role in vivo. Similar to our present report, NK cells binding the B57-NS1 tetramer had an intermediate CD56 (CD56^dim^) expression [36], consistent with the expected phenotype of a mature NK cell that expresses KIR [17,37]. A study examining NK phenotypes in subjects with recurrent HSV-2 showed no skewing of NK cells to a highly differentiated or terminal effector phenotype, suggesting HSV-2 peptides have no influence on KIR engagement, albeit the HLA-types of the study subjects were unknown [37]. As KIR3DL1 is expressed by a subset of both NK and T cells, multiple levels of immune crosstalk are plausible during HSV infection. For example, KIR3DL1 positive NK cells could be modulated by HSV-2 infection as a manifestation of trained immunity [38]. The study of large cohorts of HLA-B*57:01-positive persons with and without HSV-2 infection will be required to evaluate this. Dynamic changes in KIR3DL1-expressing NK or T cells during primary infection or reactivation of HSV-2 are also possible and could be studied using serial sampling. Our findings could permit deeper scrutiny at the HLA-KIR genotype level of the impact of HSV-2 infection on NK function.

At this time, the potential roles for viral peptide-HLA interactions with KIR3DL1-expressing lymphocytes are speculative. KIR3DL1 ligation is thought to inhibit NK cell activation. Therefore, the interaction of HLA-B*57:01 complexed to the HSV-2 RTR peptide on the surface of HSV-2 infected cells could prevent NK cell activation, and thus facilitate immune escape from NK cell host defense. Counterbalancing this, profound reduction in cell surface HLA class I expression via the inhibition of a transporter associated with antigen processing (TAP) by HSV [39] decreases HLA interactions with inhibitory KIRs and thus increases the susceptibility of infected cells to NK cell killing [40]. HLA alleles (and their peptides) differ in their dependence on TAP for transport to the cell surface [41,42] and in the degree they are impacted by virally encoded HLA-reducing genes [43]. It is, therefore, of interest to determine how strongly HSV-1 and HSV-2 inhibit HLA-B*57:01 on the cell surface. Adding complexity, CD8 T cells as well as NK cells can express KIR3DL1 (Figure 2C). Thus, B57-RTR could provide a general inhibitory signal to CD8 T cells with diverse TCRs, contributing to broad immune escape. In addition, the extracellular domains of KIR3DL1 and KIR3DS1 are largely identical. Prior work shows that viral peptide bound to HLA-B*57:01 can bind KIR3DS1 [16], albeit other data suggest KIR3DS1 binds poorly to HLA-B*57:01 [15], whereby the binding ability of either receptor is influenced by the peptide composition. As KIR3DS1 contributes to lymphocyte activation, future work on the potential interaction of HLA-B*57:01 with KIR3DS1 could reveal active NK- or T-cell sensing of HSV-infected cells.

T cells are thought to mediate abacavir toxicity in persons with an HLA-B*57:01 genotype [7]. Abacavir can occupy a pocket in the peptide-binding groove of HLA-B*57:01 to displace the C-terminal amino acid of bound peptide. This could lead to the formation of neo-epitopes that had not participated in negative selection in the thymus and, thus, auto-reactivity. Abacavir binding can also cause the loss of CD8 T-cell responses to pathogens, as recently observed for a HLA-B*57:01-restricted epitope from the gamma-herpesvirus Epstein–Barr virus (EBV) [8]. A similar observation has been made for the antibiotic flucloxacillin, which also generates hypersensitivity reactions related to CD8 T cells, albeit by a mechanism that appears to increase self protein-derived neo-epitopes by modifying lysine residues within peptide sequences [44]. Arguably, altered conformations of self peptides could also impact interactions between HLA-B*57:01 and KIR3DL1, as proposed for other KIR types [45]. For instance, if an altered epitope prevents the KIR3DL1-mediated inhibition of NK cells or KIR3DL1-positive T cells, an undesired auto-reactive response could ensue. Arguing against this, there are no reports of abacavir toxicity correlating with KIR alleles or haplotypes. The incomplete penetrance of abacavir toxicity in HLA-B*57:01-positive persons remains unexplained and could be related to a combination of KIR variation and viral infection. In our study, we included a peptide from HSV-1 orthologous to the HSV-2 RTR epitope and sequence-related peptides in human IFNL2 and IFNL3. CD8 T-cell lines cross-reacted with the HSV-1 peptide, but not the self-peptides. No change in reactivity was observed to any peptide in the presence of abacavir, suggesting that these particular HSV-2-specific CD8 T cells may not participate in abacavir-induced hypersensitivity. Since epitope-specific CD8 T cells occur as a polyclonal swarm within-person [46], studies of RTR-specific clonal T cells with defined TCRs could uncover modulation by abacavir. We have not yet examined the effect of abacavir on the interaction between HLA-B*57:01-RTR and KIR3DL1 to examine the possibility that the drug influences KIR3DL1 function.

In summary, both TCR on CD8 T cells, and the KIR3DL1 molecule, can bind to complexes of HLA-B*57:01 and a peptide epitope that we newly identified in the HSV-2 ICP22 protein encoded by gene *US1*. The specificity of binding was confirmed by the heterotopic expression of KIR3DL1 in human KIR-negative non-human primate COS-7 cells, and by blocking with the KIR3DL1-specific DX9 mAb. NK cells are known to play an important role in host defense against HSV, but further work will be required to determine if the molecular interaction reported in this paper has functional consequences for recognition or escape from cells including NK cells and KIR-expressing T cells in persons with diverse KIR haplotypes.

## Figures and Tables

**Table 1:** Peptides tested for reactivity with polyclonal HSV-2-reactive CD8 T-cell lines.

Peptide Name	Species	Gene/ORF	Protein	AA Position in Protein	Peptide Sequence
HSV2-US1 (36–45)	HSV-2	*US1*	ICP22	36–45	PSSSESEGKP
HSV2-US1 (50–59)	HSV-2	*US1*	ICP22	50–59	ESSSTESSED
HSV2-US1 (55–64)	HSV-2	*US1*	ICP22	55–64	ESSEDEAGDL
HSV2-US1 (105–114)	HSV-2	*US1*	ICP22	105–114	DASDGWLVDT
HSV2-US1 (158–167)	HSV-2	*US1*	ICP22	158–167	PASLPGIAHA
HSV2-US1 (228–236)	HSV-2	*US1*	ICP22	228–236	RTRLGPRTW
HSV2-US1 (295–304)	HSV-2	*US1*	ICP22	295–304	STSDDEISDA
HSV2-US1 (350–359)	HSV-2	*US1*	ICP22	350–359	WTSEEGSQPW
HSV2-US1 (366–375)	HSV-2	*US1*	ICP22	366–375	DTSSAERSGL
HSV1-US1 (232–240)	HSV-1	*US1*	ICP22	232–240	RaRLaPRTW *
IFNL3 (76–84)	Human	*IFNL3*	IFNL3 (IL28B)	76–84	RsRLfPRTW *
IFNL2 (76–84)	Human	*IFNL2*	IFNL2 (IL28A)	76–84	HsRLfPRTW *

* Variant amino acids different from peptide HSV2-US1 (228–236) are shown in lower case.

## Data Availability

The data presented in this study are available upon reasonable request from the corresponding author.
